# Factors influencing trustworthiness and perceived biases of medical information and genetic testing for Black and White Americans

**DOI:** 10.1371/journal.pgen.1011800

**Published:** 2025-10-31

**Authors:** Crystal Lederhos Smith, Brian Connor Stark, McKenna Kobalter, Mary Carol Barks, Mariko Nakano-Okuno, Ellen Weger Romesburg, Nita Limdi, Thomas May

**Affiliations:** 1 Washington State University, Elson S. Floyd College of Medicine, Spokane, Washington, United States of America; 2 The University of Alabama at Birmingham Heersink School of Medicine, Birmingham, Alabama, United States of America; Baylor College of Medicine, UNITED STATES OF AMERICA

## Abstract

Ensuring diversity in genomic research is crucial to address disparities in healthcare benefits experienced by Black Americans and other minority groups. Despite progress in promoting diversity, Black Americans remain underrepresented in most genetic studies, resulting in unequal access to the benefits of genetic medicine. This study investigates trusted sources of medical and genetic testing information among Black and White Americans, identifying key factors that influence trust and participation in genetic research. Using an online survey of 1,018 participants (Black Americans n = 500, White Americans n = 518), we analyzed trust and bias ratings across various sources, including medical providers, genetic counselors, and social media. Medical providers emerged as the most trusted source for both medical and genetic information across racial groups. In terms of bias, social media was viewed as most biased and medical providers as least biased across both groups. However, Black Americans reported significantly lower trust in medical providers and scientific literature compared to White Americans. Furthermore, Black Americans expressed a stronger preference for receiving medical information from individuals of the same race or ethnicity. These findings highlight the importance of tailoring communication outlets and strategies to address the specific trust concerns of underrepresented populations. Efforts to engage Black Americans in genetic research may benefit from increased involvement of medical providers and genetic counselors, improved transparency, and culturally relevant communication. By addressing these factors, the research community can work towards reducing disparities and promoting equitable access to the benefits of genetic medicine.

## Author summary

Despite progress in promoting diversity, Black Americans remain underrepresented in most genetic studies, resulting in unequal access to the benefits of genetic medicine. This study investigates trusted sources of medical and genetic testing information among Black and White Americans, identifying key factors that influence trust and participation in genetic research. Using an online survey of 1,018 participants (Black Americans n = 500, White Americans n = 518), we analyzed trust and perceived bias across various information sources. Medical providers were the most trusted source for both medical and genetic information across racial groups and social media was viewed as most biased. Black Americans reported significantly lower trust in scientific literature and medical providers, compared to White Americans, and expressed a stronger preference for receiving medical information from individuals of the same race or ethnicity. To reduce disparities and promote equitable access to the benefits of genetic medicine, scientists should work closely with medical providers and avoid blanket advertisements through media, focusing on transparency and culturally relevant communication.

## Introduction

Perhaps the greatest challenge currently facing genetic research is ensuring adequate diversity among research participants. Diversity of participants is essential both for good science and to promote equity in the realization of its benefits across all segments of society. The U.S. healthcare system has been plagued by what has been described as ‘racially divided health care’ [1] that has historically resulted in lower life expectancy and increased health problems among Black Americans - even after accounting for socioeconomic factors influencing health - compared to their white counterparts [2]. This is particularly true in the realm of genetic medicine, which sees tremendous disparity in the realization of benefits from genetic medical research, largely due to the failure to utilize available resources. For example, one study found that Black American and Hispanic patients are less likely than White patients to undergo testing for Lynch syndrome or to be recommended for genetic evaluation [3]. Another study found that 58.8% of Black American breast cancer patients who met National Comprehensive Cancer Network (NCCN) genetic testing guidelines did not receive testing as part of routine care [4].

The above underutilization of resources might be accounted for through individual decisions to avoid participation, or by failure of health practitioners to equally apply the fruits of research to all populations [5]. In both cases, trust, summarized as the optimistic acceptance of a vulnerable situation in which the truster believes the trustee will care for the truster’s interests [6], and perceived bias (supporting or opposing a particular person or thing in an unfair way, because of allowing personal opinions to influence your judgment) [7] are likely at the heart of these challenges. At the level of individual decisions to forego available resources, our own prior qualitative research on willingness to utilize medical resources among Black American populations in particular has identified an intertwined fear of racial discrimination and medical mistrust as a main contributing factor to underutilization [8]. In terms of health providers applying research findings in an equitable manner to all patient populations, the Kaiser Family Foundation has found that the challenge of provider application of the fruits of research to all populations is in part perpetuated by low participation rates in research among minority populations, obscuring applicability of findings for populations not represented [5]. This is particularly true for genetic research, where inheritance of genes makes ancestral backgrounds particularly relevant to application of research findings [9]. This, too, is significantly influenced by trust: numerous studies including Black American participants have illustrated the interest in genetic research if trust is established [10–13]. Thus, the challenges posed by lack of diversity among research participants is likely not due to a lack of interest in participation, but rather to issues of trust.

The importance of addressing these disparities through increased participation of racial and ethnic minorities in genetic research has been recognized for some time [14–16], and progress has been made in the promotion of diversity in recruitment for genomic research for several racial demographics [17–20]. Additionally, recruitment strategies employed by multiple national and regional networks and initiatives [30–33] have made progress through utilization of expanded outreach to under-represented communities. For example, the Alabama Genomic Health Initiative (AGHI) utilized “pop-up clinics” in areas with high Black American populations, and “All of Us” has emphasized outreach to specific communities. To address mistrust and promote recruitment of diverse participants, genomic research initiatives such as *All of Us*, eMERGE, and others have placed priority on assuring privacy and promoting transparency [21,22]. Their results show that, as of June 2022, 20.5% of *All of Us* participants are Black or African Americans (whereas Black Americans make up 11.7% of the U.S. population), and 16.3% are Hispanic or Latino (as opposed 16.8% of the U.S. population) [23], thus, the *All of Us* project has successfully acquired increased representation of some racial groups. Nonetheless, recruiting from these communities has remained challenging for genomic research overall, with minimal improvements in the diversity of genomic data, especially among Black Americans [24,25]. Because of past underrepresentation, more improvement is needed to attain genomic health equity, with clearer understanding of what constitutes effective recruitment strategies for underrepresented groups [22]. Additionally, community-engagement efforts to attain equitable representation are new and their effectiveness is yet to be fully known (e.g., the second iteration of the BabySeq Project began in 2023) [26]. To be effective, recruitment goals and reasoning must be communicated in a manner that resonates with the specific diverse communities targeted. Currently, there is little variation in communication about genetic medicine and research across racial groups or targeting of communication strategies specific to minorities with lower rates of participation.

The research presented herein aimed to determine whether there are statistically significant differences in trusted sources of a) medical and b) genetic testing information, with the goal of identifying effective sources of information to advance effective communication and promote utilization of genomic resources and/or recruitment of diverse participants in genomic research. We did this by conducting a nationwide survey on trusted sources of information, both in terms of spokespersons and in terms of venues for relaying information to minority populations, with a focus on Black Americans. Additionally, we aimed to identify the most influential factors impacting trust, in an effort to improve communication and increase Black American engagement.

## Subjects and methods

### Ethics statement

This study has been determined to be exempt by the Washington State University Institutional Review Board. (IRB #: 20142) Formal written consent was collected from all individual participants by the online survey platform Qualtrics, as is their standard procedure prior to study participation and participants were presented with a consent form for the study so they could learn about the researchers involved, the research objective, what information was being collected, and how the data would be used.

Participant recruitment was conducted through Qualtrics recruitment platform. Inclusion criteria for the online survey included: U.S. adult, aged 18 or older, and self-identifying as non-Hispanic Black American or White American. Quality check measures were used to eliminate duplicate responses and responders who answered in patterns, provided illogical patterns of answers, or sped through the survey in less than a third of median of the overall survey duration. Participation in the online survey took participants an average of just over 10 minutes. After consenting, participants completed questions about their demographics, experience with genetic screening, and ratings of trusted and biased sources of information. Responses to all non-demographic survey questions were required. They also entered their preferred email address for receipt of a virtual incentive gift card worth $10 USD.

To enhance sample description and review representation from potentially different perspectives of participants in differing U.S. regions, participants were asked to self-identify the region of the U.S. where they lived as part of their demographics. Participants also responded to items asking them whether they had participated in genetic testing, and if yes, what kind (ordered by doctor, direct to consumer, testing for a research study). They reported on whether they planned to participate in genetic testing in the future and they were asked to identify their most trusted and second most trusted sources of medical and genetic information and to rate how trustworthy (or in separate tables how biased) they felt each of several listed sources of medical or genetic information were. Response options ranged from 1 = not trustworthy at all to 5 = very trustworthy for ratings of trust and 1 = not biased at all to 5 = very biased, for ratings of bias. Participants also answered questions asking whether they would prefer to receive medical/genetic information from someone of the same race/ethnicity or gender as them. Participants answered whether they had concerns with genetic testing and, if they did, they were asked to complete an open text field describing the concerns. Finally, participants completed an open text field asking them the best way to engage more people in genetic testing.

Demographic information from participants was examined using t-tests and chi-square tests, across the Black American and White American groups, to determine whether there were significant differences in demographics that should be controlled for (Table 1). Analysis of covariance (ANCOVA) was used to compare ratings of trust and bias across Black American and White American races for medical provider, major health organizations, known people in the medical field, scientific literature, family, friends, internet search, religion/faith, news, people at church, sports coaches, TV shows, and advertisements and logistic regression was used to examine differences in the odds of preferring to receive medical/genetic information from a person of the same race/ethnicity across Black American and White American races. Both analyses controlled for demographic variables that were significantly different with bivariate analyses between the races. No missing data techniques were employed due to minimal missing data. Standard analysis procedures were followed for our analyses, including listwise deletion for participants with missing data.

**Table 1:** Demographic information.

Variable	Categories	Black American	White American	Total	p-value
**Gender**	Man	120 (23.9)	132 (25.6)	252 (24.8)	0.560
	Women	380 (75.5)	376 (73.0)	756 (74.3)	
	Non-binary	2 (0.4)	3 (0.6)	5 (0.5)	
	Transgender	–	2 (0.4)	2 (0.2)	
	None of these describe me	1 (0.2)	2 (0.4)	3 (0.3)	
**Age**	Mean (SD)	42.08 (15.77)	57.09 (16.31)	49.68 (17.71)	<0.001
**Current Employment Status**	Full-time	201 (40.0)	144 (28.0)	345 (33.9)	< 0.001
	Part-time	87 (17.3)	63 (12.2)	150 (14.7)	
	Homemaker	26 (5.2)	33 (6.4)	59 (5.8)	
	Student	32 (6.4)	9 (1.7)	41 (4.0)	
	Disabled	43 (8.5)	38 (7.4)	81 (8.0)	
	Unemployed	62 (12.3)	55 (10.7)	117 (11.5)	
	Contract or Gig	6 (1.2)	6 (1.2)	12 (1.2)	
	Retired	34 (6.8)	153 (29.7)	187 (18.4)	
	Prefer not to answer	6 (1.2)	3 (0.6)	9 (0.9)	
	Other	6 (1.2)	11 (2.1)	17 (1.7)	
**Average Monthly Personal Income**	<$500	69 (13.7)	38 (7.4)	107 (10.5)	< 0.001
	$501 - $900	70 (13.9)	31 (6.0)	101 (9.9)	
	$901 - $1300	101 (20.1)	55 (10.7)	156 (15.3)	
	$1301 - $1700	61 (12.1)	76 (14.8)	137 (13.5)	
	$1701 - $2100	61 (12.1)	58 (11.3)	119 (11.7)	
	>$2100	140 (27.8)	255 (49.5)	395 (38.8)	
**Current Marital Status**	Single	265 (52.7)	109 (21.2)	374 (36.7)	< 0.001
	Married or Cohabitating with Partner	145 (28.8)	238 (46.2)	374 (36.7)	
	Divorced or separated	63 (12.5)	112 (21.7)	383 (37.6)	
	Widowed	23 (4.6)	54 (10.5)	175 (17.2)	
	Other	2 (0.4)	1 (0.2)	77 (7.6)	
	Prefer not to answer	3 (0.6)	–	3 (0.3)	
**Highest Education Level**	Less than high school	17 (3.4)	9 (1.7)	26 (2.6)	< 0.001
	High school or equivalent	156 (31.0)	99 (19.2)	255 (25.0)	
	Technical or occupational certificate	24 (4.8)	21 (4.1)	45 (4.4)	
	Some college	117 (23.3)	124 (24.1)	241 (23.7)	
	Associate degree	59 (11.7)	58 (11.3)	117 (11.5)	
	Bachelor’s degree	89 (17.7)	123 (23.9)	212 (20.8)	
	Master’s degree	29 (5.8)	68 (13.2)	97 (9.5)	
	Doctorate, PhD, MD, or equivalent	11 (2.2)	12 (2.3)	23 (2.3)	
	Other	1 (0.2)	1 (0.2)	2 (0.2)	
**Region**	Northeast	109 (21.7)	105 (20.4)	214 (21.0)	< 0.001
	Southwest	109 (21.7)	62 (12.0)	171 (171.0)	
	Northwest	50 (9.9)	86 (16.7)	136 (13.4)	
	Southeast	165 (32.8)	152 (29.5)	317 (31.1)	
	Midwest	70 (13.9)	110 (21.4)	180 (17.7)	

Note: p-values listed are resultant of t-tests for continuous variables and chi-square tests for categorical variables.

Code, implemented in R, for calculating the values reported in Tables 3 and 4 follows:

**Table 2:** Frequency distribution of most trusted source of information by race.

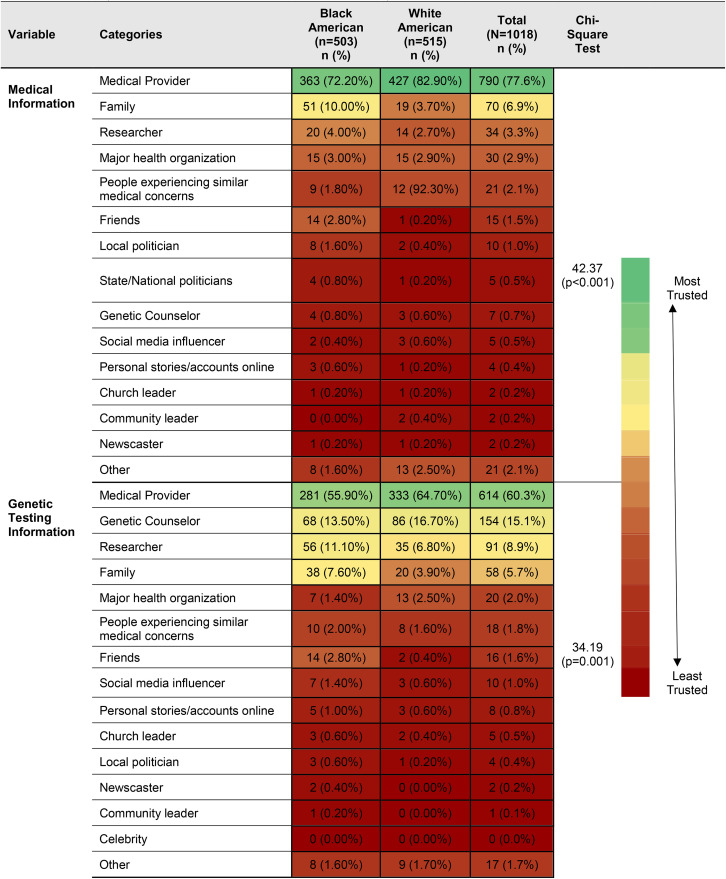

**Table 3:** Ratings of trust in medical information and genetic testing information and their differences across Black and White American races.

Trust in Medical Information					
	Black American n = 503	White American n = 515	F Statistic	p-value	Total N = 1018
**Medical Provider**	4.50 (0.88)	4.75 (0.59)	7.26	**0.007**	4.63 (0.75)
**Known People in the Medical Field**	3.86 (1.13)	3.96 (0.91)	0.20	0.652	3.91 (1.02)
**Major Health Organizations**	3.96 (1.11)	3.85 (1.20)	1.63	0.201	3.90 (1.16)
**Family**	3.74 (1.06)	3.85 (0.89)	2.31	0.128	3.80 (0.98)
**Web MD**	3.62 (1.15)	3.73 (0.97)	1.05	0.305	3.68 (1.07)
**Scientific Literature**	3.49 (1.19)	3.78 (1.03)	8.35	**0.003**	3.64 (1.12)
**Friends**	3.37 (1.09)	3.52 (0.87)	3.16	0.075	3.45 (1.13)
**Internet Search**	3.32 (1.16)	3.25 (1.07)	0.40	0.528	3.29 (1.11)
**Religion/Faith**	3.21 (1.32)	2.61 (1.31)	43.11	**≤0.001**	2.91 (1.35)
**News**	3.01 (1.21)	2.70 (1.15)	15.14	**≤0.001**	2.85 (1.19)
**People at Church**	2.87 (1.23)	2.49 (1.11)	20.48	**≤0.001**	2.67 (1.18)
**Sports Coaches**	2.72 (1.22)	2.46 (1.08)	0.938	0.333	2.59 (1.16)
**TV Shows**	2.71 (1.16)	2.42 (1.09)	10.18	**0.001**	2.56 (1.13)
**Advertisements**	2.69 (1.16)	2.25 (1.05)	20.38	**≤0.001**	2.47 (1.13)
**Social media**	2.48 (1.23)	2.04 (2.04)	10.09	**0.001**	2.26 (1.20)
**Trust in Genetic Testing Information**					
	**Black American** n = 503	**White American** n = 515	**F** **Statistic**	**p-value**	**Total** N = 1018
**Medical Provider**	4.26 (0.95)	4.56 (0.71)	9.19	**0.002**	4.41 (0.86)
**Known People in the Medical Field**	3.69 (1.19)	3.74 (1.22)	0.26	0.611	3.72 (1.21)
**Major Health Organizations**	3.60 (1.15)	3.77 (0.97)	0.00	0.987	3.69 (1.06)
**Family**	3.46 (1.23)	3.76 (1.09)	1.03	0.310	3.61 (1.16)
**Web MD**	3.53 (1.15)	3.61 (1.01)	1.39	0.238	3.57 (1.08)
**Scientific Literature**	3.41 (1.19)	3.55 (1.01)	6.12	**0.013**	3.48 (1.10)
**Friends**	3.12 (1.19)	3.25 (1.01)	1.43	0.232	3.19 (1.10)
**Internet Search**	3.16 (1.22)	3.04 (1.16)	0.95	0.329	3.10 (1.19)
**Religion/Faith**	2.91 (1.30)	2.48 (1.22)	27.14	**≤0.001**	2.69 (1.28)
**News**	2.82 (1.23)	2.51 (1.15)	9.56	**0.002**	2.66 (1.10)
**People at Church**	2.70 (1.24)	2.39 (1.13)	15.71	**≤0.001**	2.55 (1.19)
**Sports Coaches**	2.60 (1.19)	2.29 (1.02)	2.36	0.124	2.45 (1.11)
**TV Shows**	2.56 (1.21)	2.31 (1.05)	12.08	**≤0.001**	2.43 (1.14)
**Advertisements**	2.63 (1.18)	2.11 (1.06)	25.71	**≤0.001**	2.37 (1.15)
**Social media**	2.35 (1.25)	1.84 (1.06)	9.45	**0.002**	2.09 (1.19)

Note: Numbers are presented in Mean (SD) format. Bold numbers indicate significant comparisons (*p* ≤ 0.05), controlling for demographics that were significantly different across races (i.e., age, employment status, monthly income, marital status, educational level, and region of residence). Ratings were based on a scale of 1 = not trustworthy to 5 = very trustworthy. P-values and F-statistics from analysis of covariance (ANCOVA) comparing trust in medical and genetic testing for Black and White American races.

**Table 4:** Ratings of bias in medical information and genetic testing information and their differences across Black and White American races.

Bias in Medical Information					
	Black American n = 503	White American n = 515	F Statistic	p-value	Total N = 1018
**Social media**	3.52 (1.19)	4.00 (1.10)	12.42	**≤0.001**	3.76 (1.17)
**Advertisements**	3.41 (1.15)	3.91 (1.11)	17.41	**≤0.001**	3.66 (1.15)
**TV Shows**	3.37 (1.12)	3.66 (1.07)	7.95	**0.004**	3.51 (1.10)
**News**	3.28 (1.18)	3.57 (1.08)	7.18	**0.007**	3.42 (1.13)
**Sports Coaches**	3.22 (1.10)	3.34 (1.01)	0.151	0.697	3.28 (1.05)
**Religion/Faith**	3.15 (1.27)	3.36 (1.22)	7.40	**0.006**	3.26 (1.14)
**People at Church**	3.16 (1.19)	3.33 (1.11)	4.30	**0.038**	3.24 (1.16)
**Internet Search**	3.11 (1.14)	3.08 (1.11)	0.113	0.736	3.10 (1.12)
**Friends**	3.07 (1.18)	2.81 (1.11)	2.82	0.092	2.94 (1.15)
**Family**	3.02 (1.26)	2.72 (1.19)	0.748	0.387	2.87 (1.24)
**Major Health Organizations**	2.85 (1.35)	2.61 (1.34)	1.43	0.230	2.73 (1.35)
**Scientific Literature**	2.88 (1.20)	2.51 (1.15)	15.93	**≤0.001**	2.69 (1.19)
**Known People in the Medical Field**	2.82 (1.23)	2.52 (1.06)	1.77	0.182	2.67 (1.16)
**Web MD**	2.80 (1.24)	2.51 (1.06)	5.10	**0.024**	2.65 (1.16)
**Medical Provider**	2.64 (1.37)	2.15 (1.24)	4.34	**0.037**	2.39 (1.32)
**Bias in Genetic Testing Information**					
	**Black American** n = 503	**White American** n = 515	**F Statistic**	**p-value**	**Total** N = 1018
**Social media**	3.43 (1.28)	3.91 (1.17)	6.29	**0.012**	3.68 (1.25)
**Advertisements**	3.41 (1.20)	3.81 (1.13)	7.39	**0.006**	3.61 (1.18)
**TV Shows**	3.28 (1.17)	3.63 (1.01)	10.55	**0.001**	3.45 (1.10)
**News**	3.25 (1.18)	3.47 (1.09)	2.37	0.123	3.36 (1.14)
**Sports Coaches**	3.17 (1.22)	3.33 (1.21)	4.38	**0.036**	3.25 (1.21)
**Religion/Faith**	3.13 (1.16)	3.32 (1.15)	5.64	**0.017**	3.23 (1.16)
**People at Church**	3.11 (1.15)	3.34 (1.05)	6.24	**0.012**	3.23 (1.10)
**Internet Search**	3.10 (1.20)	3.07 (1.08)	0.031	0.8585	3.08 (1.14)
**Friends**	2.96 (1.19)	2.74 (1.06)	4.01	**0.045**	2.85 (1.13)
**Family**	2.93 (1.25)	2.63 (1.20)	1.37	0.241	2.78 (1.23)
**Major Health Organizations**	2.85 (1.27)	2.60 (1.10)	2.20	0.138	2.72 (1.20)
**Scientific Literature**	2.82 (1.33)	2.61 (1.35)	14.16	**≤0.001**	2.72 (1.34)
**Known People in the Medical Field**	2.86 (1.25)	2.54 (1.08)	2.99	0.083	2.70 (1.18)
**Web MD**	2.83 (1.25)	2.46 (1.13)	1.45	0.227	2.65 (1.20)
**Medical Provider**	2.60 (1.38)	2.08 (1.25)	8.95	**0.002**	2.34 (1.33)

Note: Numbers presented in Mean (SD) format. Bold numbers indicate significant comparisons (*p* ≤ 0.05), controlling for demographics that were significantly different across races (i.e., age, employment status, monthly income, marital status, educational level, and region of residence). Ratings were based on a scale of 1 = not biased to 5 = very biased. P-values and F-statistics from analysis of covariance (ANCOVA) comparing bias in medical and genetic testing for Black and White American races.

library(car)

ancova1 <- aov(MT1 ~ Race + Age + Employment_Status + Monthly_Income + Marital_Status + Education + Region, data = data_emerge)

Anova(ancova1, type=“III”)

Data used for the analyses are available (see S1 Data). Specifically, data used to calculate trust in medical information can be found in columns R (Q25_1) through AF (Q25_15), and genetic testing information can be found in columns BD (Q38_1) through BR (Q38_15). Specifically, data used to calculate bias in medical information can be found in columns AG (Q27_1) through AU (Q27_15), and genetic testing information can be found in columns BS (Q39_1) through CG (Q39_15).

## Results

The survey was completed by N = 1,018 participants, 74.3% of which (n = 756) identified as women. Average age of participants was 49.68 years old (SD = 17.71). See Table 1 for full demographic information and p-values comparing responses between Black Americans and White Americans. Missing data was minimal and limited to demographics responses due to a set requirement in the survey that all non-demographic items required a response. Three respondents chose not to select an answer reporting their personal income and three chose not to select an answer reporting their marital status. Although all open-ended questions required a response, n = 398 (39%) responses to the question, *“What information, actions, or services would help you trust a medical provider?”* were not included in our analysis. These responses were not included due to participants not answering the question with responses like “*none,*” “*not applicable*,” or “*I don’t know*;” other responses were not included in analysis due to being unrelated to the question or indecipherable by coders. Participants were required to provide information on their race to ensure they met inclusion requirements.”

### Demographics

**Fig 1:**
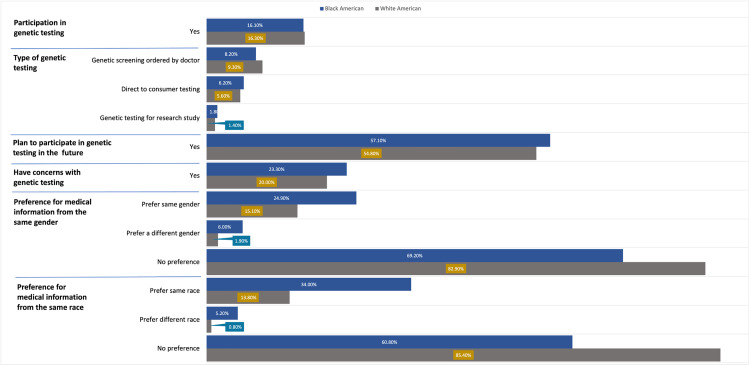
Genetic testing participation and medical information preferences across Black and White American races.

There were no significant differences across the two groups in gender identity, previous participation in genetic testing or testing types, plan to have genetic testing in the future, or concern with genetic testing. Just over 16% of our sample had previously participated in genetic testing. Of these, 54% (n = 89) had the testing ordered by a doctor, 67% (n = 60) used direct-to-consumer testing, and 27% (n = 16) participated in a research study where they were tested. In response to a question asking whether participants planned to have genetic testing done in the future, 55.9% said yes and 44.1% reported that they either did not plan to or had already participated in genetic testing. In response to a question asking whether participants had concerns with genetic testing, 78.4% (n = 798) replied that they did not. See Fig 1 for additional detail on these descriptives.

Significant differences between the groups were found across age, employment status, monthly income, marital status, educational level, and region of residence. White Americans were, on average, 15 years older than Black Americans in our sample. In comparison, Black Americans in the general US population are, on average, 10 years younger than our sample, and White Americans in the general US population are, on average, 16 years younger than our sample [27–29]. More Black Americans selected single (52.7% vs 21.2%), whereas more White Americans selected married or cohabitating with a partner (21.7% vs 12.5%) and divorced/separated or widowed (32.2% vs 17.1%). Nearly half of the White American sample had a personal monthly income over $2100, whereas only 27.8% of Black Americans selected this category. In addition, Black Americans selected the category of making less than $500 per month nearly twice as frequently as White Americans (13.7% vs 7.4%, respectively). Additional detail can be found in Table 1. Demographic information.

### Most Trusted Sources of information

In response to questions asking respondents to select their most trusted and second most trusted sources of medical and genetic testing information, medical providers were identified as the most trusted source of medical information for 72.2% (n = 363) of Black Americans and 82.9% (n = 427) of White Americans. The second most frequently identified highly trusted source of medical information lagged substantially behind, with 10.1% (n = 51) of Black Americans and 3.7% (n = 19) of White Americans selecting family as a trusted source of medical information. Medical providers were also identified as the most trusted source of genetic testing information, with 55.9% (n = 281) of Black Americans and 64.7% (n = 333) of White Americans making this selection. Genetic counselors were identified second most frequently as the most trusted sources of genetic testing information for both races, with 13.5% (n = 68) of Black Americans and 16.7% (n = 86) of White Americans selecting them. See Table 2 for a full list of most trusted sources of medical and genetic testing information.

### Numerical ratings of trust

In response to a separate question asking participants to numerically rate how much they trusted a series of sources of information, overall, ratings of trust in medical and genetic testing information across Black Americans and White Americans were highest for medical providers and lowest for social media. Second and third most highly rated trusted sources of information were known people in the medical field (family or friends in the medical field) and major health organizations, wherein White Americans rated known people in the medical field higher than major health organizations for medical information and the opposite for genetic testing information (they also rated trust in family for medical information similarly to their ratings of major health organizations). Black Americans rated major health organizations more highly than known people in the medical field for medical information, and the opposite for genetic testing information (although the ratings were similar, and we did not run statistical comparisons within group). Second and third lowest rated sources of genetic testing information for Black Americans were TV shows and sports coaches, respectively. For White Americans the second and third lowest rated sources of genetic testing information were advertisements and sports coaches, respectively. For medical information, the second and third lowest rated sources were advertisements and TV shows, respectively, for both races. For additional detail on ratings of trust see Table 3.

Results from our statistical comparisons, which controlled for demographic variables with significant differences between groups at baseline, indicated that there were significant differences in ratings of trust for both medical information and genetic testing information for 8/15 categories assessed. These included medical providers, scientific literature, religion/faith, news, people at church, TV shows, advertisements, and social media. Although categories were the same for significant differences, ratings within these categories differed across medical information and genetic testing information. See Table 3 for a full comparison, including p-values and f-statistics from the tests.

### Qualitative feedback on trust

In response to a free text survey question asking, “What information, actions or services would help you trust a medical provider?”, participants emphasized the importance of a provider’s level of education and experience, representing 18% (n = 50) of Black American and 26% (n = 88) of White American responses. Provider reputation was an important consideration for some participants, representing approximately 20% of responses for both Black Americans (n = 58) and White Americans (n = 68); participants reported relying on recommendations and online reviews to find medical providers that they could trust. Participants also cited the importance of provider transparency and demonstrated lack of bias; for example, participants trusted providers that protected their personal information and disclosed any conflicts of interest (Black Americans 16%, n = 44; White Americans 19%, n = 66). Numerous participants emphasized the importance of medical providers presenting thorough and honest information about treatment options in building trust, representing 45% (n = 123) of Black Americans and 25% (n = 86) of White Americans. Before receiving medical services, participants stated that they would trust providers whose information about treatment options included the risks, benefits, and side effects, evidence from the scientific literature, and therapeutic relevance to the patient. Good communication skills, showing empathy, and taking patient concerns seriously were also cited as important components of establishing trust with a provider (Black Americans 13%, n = 36; White Americans 17%, n = 57). A medical provider’s ability to relate to the patient also increased provider-patient trust; three participants stated that the provider being of the same gender and two stated that the provider being of the same race was important.

### Perceptions of bias

In response to a question asking participants to numerically rate how “biased/unbalanced/agenda driven” they felt a series of sources of medical and genetic testing information were, ratings for both Black Americans and White Americans were highest for social media and lowest for medical providers, suggesting that participants felt social media was very biased and medical providers were not very biased. The second and third most highly rated sources of biased information were advertisements and TV shows, respectively, for both groups. White Americans consistently rated sources of medical and genetic testing information as more biased than Black Americans did, across these most highly rated categories (Table 4).

Statistical comparisons, which controlled for demographic variables with significant differences between groups at baseline, indicated that there were significant differences in ratings of bias in 9/15 categories assessed for medical information and 9/15 categories for genetic testing information. Significant differences between races, in ratings of bias for both medical and genetic testing information, were found for social media, advertisements, TV shows, religion/faith, people at church, scientific literature, and medical providers, with Black American participants rating scientific literature and medical providers as significantly more biased than White Americans and White Americans rating social media, advertisements, TV shows, religion/faith, and people at church significantly higher (more biased) than Black Americans rated them. Medical information in the news and genetic testing information conveyed by sports coaches and friends were rated significantly higher (more biased) by White Americans than Black Americans and Web MD was rated significantly higher (more biased) by Black Americans than White Americans. See Table 4 for a full comparison of ratings of bias.

### Receiving information from someone of the same or a different race

While the majority of participants across the groups had no preference as to whether they received medical/genetic testing information from someone of the same or a different race as they were (60.8% [n = 306] of Black Americans and 85.4% [n = 440] of White Americans),

significantly more Black Americans than White Americans preferred having a person of the same race/ethnicity provide the information (34% vs 13.8% of White Americans), with logistic regression results showing that the odds of Black Americans indicating that they preferred to receive the information from a provider of the same race/ethnicity were 2.78 times that of White Americans (p < 0.001, Wald = 30.34, exp(B)=2.78). Additionally, though the percentages were small, more Black Americans than White Americans preferred having a person of a different race provide the information (5.2% vs 0.8% of White Americans), with logistic regression results showing that the odds of Black Americans indicating that they preferred to receive the information from a provider of the same race/ethnicity were 2.78 times that of White Americans (p = 0.04, Wald = 4.29, exp(B)=3.27).

## Discussion

To fairly distribute the benefits from genetic medicine, better participation is needed from minority groups (particularly Black Americans), both as research participants and as consumers of genetic medicine resources and interventions. While progress has been made in this area, [30–33] as discussed above, this progress has been uneven, and diversity of participants continues to be an ongoing challenge. Further progress will require that we move beyond expanded outreach and address issues of distrust that negatively influence motivation to participate among underrepresented individuals. Communication of information that might improve each type of participation will only be effective if the information is perceived as trustworthy.

The goal of this research was to identify effective sources of information to advance communication and promote utilization of genomic resources and/or recruitment of diverse participants in genomic research. The results of our study provide valuable guidance that can be utilized by both researchers and clinicians to better communicate information in ways that is perceived as trustworthy. Below, we highlight what we consider to be the most important guidance taken from our study results, while recognizing that others may find alternative aspects more relevant to their specific research or clinical needs.

Most importantly, there are clear sources for conveyance of information that are trusted across the participant groups that were included in this study. Medical providers, while not universally trusted, are consistently identified as the most trusted source of information for both Black and White populations surveyed. They are the only source cited by at least 20% of participants as a trusted source of information in either category (medical or genetic testing), and the only source cited by more than 10% of participants as a trusted source of medical information. Thus, for both groups, medical providers should be the bedrock for communication.

While our study identified several similarities across trust and bias in sources of information (e.g., trust in medical providers and bias in social media), differences in numerical ratings and rankings varied by race and information type (medical vs. genetic testing). Additionally, participant ratings of bias in medical information versus genetic testing information differed between racial groups across the following categories: news, sports coaches, friends, and Web MD, suggesting that both trust and bias are context dependent, depending on the type of information being conveyed. As such, communications strategies should be tailored to reflect these nuances.

Although ratings for trust varied across our two groups, similarities arose that provide insights into enhancing engagement and communication. Aside from medical providers, results consistently point to a small handful of most trusted sources, including family, researchers, genetic counselors, known people in the medical field, and major health organizations. Improvement in advancing effective communication and promoting utilization of genomic resources and/or recruitment of diverse participants in genomic research should focus on these trusted sources.

Qualitative feedback on trust in medical providers emphasized the importance of a provider’s level of education and experience, reputation, transparency and demonstrated lack of bias, and presentation of thorough and honest information about treatment options. These findings align with our previous research suggesting that both Black and White Americans focus on professional experience/training, relationships, objectivity, and ability to cross-check information [8]. These are components that promote trust in their medical providers – their most trusted sources of information – thus, it relationship building and communications with other trusted sources (e.g., researchers and major health organizations) should also focus on these components.

Related to the above concerns, the current study found that although the majority of both Black and White participants expressed no preference for the race of the person providing medical information, a significant difference existed for the share of participants expressing a preference for information to be provided by a person of the same race (34% of Black participants versus 13.8% of White participants). While recent studies have shown mixed results regarding benefits of patient-provider racial concordance in general [34–37], this may be an area in need of a more nuanced examination. For example, some recent work has suggested that racial or ethnic concordance is related with health care satisfaction but is not related to other more tangible medical outcomes [38]. Additionally, preference for patient-provider concordance has been noted specifically in studies focused on Black patients [39], greater perceived quality of care has been identified for older Black patients with racial concordance with their provider [40], and improved decision-making outcomes have been found specifically for Black patients [41], thus, speaking to racial concordance may be too general – it may be more appropriate to focus on racial concordance for specific groups of people with more targeted outcomes in order to appropriately utilize this burgeoning information.

Integration and application of the multiple aspects of these results could manifest in several ways. Examples include, 1) hosting family centered workshops with information explaining genomic testing in simple, relatable terms, using trusted messengers who reflect the community’s background, particularly for Black Americans. Key to these workshops would be transparency, addressing concerns about privacy, data use, discrimination, clearly explaining who has access to patient data, how it is stored and protected, and what it will be used for immediately and in potential future applications. 2) Co-designing informational products, research studies, and research dissemination with patients/participants of genomic testing and their families and communities. This collaborative approach will help ensure that education and dissemination efforts are relevant, culturally responsive, and meaningful. Finally, researchers and representatives from health organizations should be visible and approachable to the communities they serve. Hosting open houses, engaging with communities, and intentionally highlighting motivations for their work in an effort to promote transparency and establish themselves as neighbors and allies, will in turn increase trust and engagement.

### Implications

Research indicates that medical mistrust has been linked to many aspects of diminished health and a lack of self-promoting health behaviors such as health screenings in the Black American community [42] and White American community alike [43]. Our study focused on establishing a deeper understanding of each of these areas of trust and bias and their similarities and differences across different races and marginalized subgroups of people, in order to move engagement of underserved and underrepresented populations forward.

Key takeaways from our study include:

- Medical providers are easily the most trusted source of information for both general medical information and for genetic testing and research. Medical professionals, then, should be the bedrock of strategies to convey information about all medical research and clinical interventions.
- Many sources of trusted information and of perception of bias differed both between the race of the participant surveyed, and for the specific type of information conveyed (general medical versus genetic testing). Therefore, communication strategies, sources and materials should be customized for the type(s) of information being conveyed, and for the target population of information conveyance. Specifics of this customization should match the particulars of a given proposed research project or health intervention campaign to the specifics of the outcomes we detail in Tables 2 [4].
- Although the majority of the participants surveyed expressed no preference for the race of the person conveying information, the frequency of expressed preference for information conveyed by a person of the same race was much higher among Black participants than White. This suggests that although it is not always necessary for the person communicating with Black populations to be of the same race, it will be important for a significant portion of this population.

It is our hope that the results of this study, and in particular the details of the specific levels of trust and bias identified for both general medical information and for genetic testing, will be informative for both research studies recruiting diverse subjects, and for health intervention campaigns attempting to reach underserved populations. As with many areas of healthcare, the applicability of our study results will require careful consideration of the specific circumstances of individual research projects, clinical interventions, and participants or consumers. With this caveat in mind, we hope that the specific findings outlined in our study results will enable both researchers and health intervention campaigns to communicate more effectively.

### Limitations

Our Black American sample was significantly different from our White American sample on several demographics, including being younger, having lower income, and containing a higher percentage of people who were single. Although we controlled for each of the significant differences in our analyses, this may have impacted the outcomes of our study in ways we were unable to control for.

## Supporting information

S1 Appendix Questions presented to survey respondents. (DOCX)

S1 Data Deidentified dataset. (XLSX)
