# Supplementary material for: Factors influencing trustworthiness and perceived biases of medical information and genetic testing for Black and White Americans
Source: PLoS Genet. 2025 Oct 31;21(10):e1011800. doi: 10.1371/journal.pgen.1011800 (PMC12591488; doi:10.1371/journal.pgen.1011800)
Supplement: S1 Appendix — (DOCX) [file pgen.1011800.s001.docx]

| S1 Appendix. Questions presented to survey respondents |
| --- |
| 1. **Where would you get medical/health information? (For example: information on diseases, medical procedures, or medications)**   [Select all that apply]: |
| - My medical provider (e.g., primary care provider I see, or medical specialist I see) - People I personally know who are in the medical field - Family - Friends - Sports coaches - Professional athletes - People at church (leader/pastor or church community members) - I listen to my own heart/faith - Commercials or advertisements - News (on TV, radio, newspaper, online) - TV shows or podcasts (not including news stations) - Social media - Internet (e.g., Google) search - Web MD or similar health information website - Scientific literature - Major health organizations (e.g., World Health Organization, National Institute of Health, Mayo Clinic, CDC) - Community/Local organizations (e.g., local health department, governor’s office, regional medical association) - Other (Please describe) |
| 1. **Please select all social media options that you use as sources of health information:** |
| - Instagram/Threads - Twitter/X - Facebook/Meta - YouTube - TikTok - Pinterest - Snapchat - LinkedIn - Other |
| Questions 3-4 below were presented with Question response list #1  **3. Who would you trust the most for medical/health information?**  [Select your #1 and #2 most trusted source]  **4. Who would you trust the most for information on genetic screening?**  [Select your #1 and #2 most trusted source]  (Question Response List #1:)   - - - - My medical provider (e.g., primary care provider I see, or medical specialist I see)       - Researcher       - Local politician       - State/National politicians       - Friends       - Family       - Church leader       - Community leader       - Personal stories/accounts online       - People experiencing similar medical concerns       - Major health organization spokesperson       - Social media influencer       - Celebrity (e.g., actor/actress)       - Newscaster       - Genetic counselor       - Other |
| Questions 5-9 below were presented with Question response list #2   1. **How trustworthy are each of these in providing medical information?** 2. **How biased/unbalanced/agenda driven are each of these in providing medical information?** 3. **Where would you get information on genetic screening? [Select all that apply]** 4. **How trustworthy are each of these in providing information on genetic screening?** 5. **How biased/unbalanced/agenda driven are each of these in providing information on genetic screening?**     (Question Response List #2:)   - - - Social media (e.g., Instagram, Twitter, Facebook, YouTube, TikTok)     - Internet (e.g., Google) search     - Scientific literature     - Religion/Faith     - People I personally know who are in the medical field     - Web MD     - Major health organizations (e.g., World Health Organization, National Institution)     - My medical provider     - Family     - Friends     - TV shows or podcasts (not including news stations)     - Sports coaches     - People at church (leader/pastor or church community members)     - Commercials or advertisements     - News (on TV, radio, newspaper, online) |
